# Granulomatous gingival manifestation in Melkersson Rosenthal syndrome: a case report

**DOI:** 10.11604/pamj.2023.44.72.22704

**Published:** 2023-02-06

**Authors:** Mouna Snoussi, Faten Frikha, Hela Mnif, Donia Chebbi, Sameh Marzouk, Tahya Boudawara, Zouhir Bahloul

**Affiliations:** 1 Department of Internal Medicine, Hedi Chaker Hospital, Medical School of Sfax, Sfax, Tunisia,; 2 Laboratory of Anatomo-Pathology, Habib Bourguiba Hospital, Sfax, Tunisia

**Keywords:** Facial edema, facial palsy, fissured tongue, Melkersson Rosenthal syndrome, case report

## Abstract

Melkersson-Rosenthal syndrome is a rare condition of unknown etiology. It is characterized by a classical triad of symptoms: relapsing facial and lip swelling, facial palsy and a fissured tongue. We report the case of a 29-year-old female patient who presented with the above-mentioned symptoms of Melkersson-Rosenthal syndrome. However, clinical examination revealed an exceptional manifestation, which is the gingival hyperplasia. The symptoms were partially managed with systemic steroids and surgical resection of gingival hyperplasia. The most significant finding to emerge from our case is that gingival enlargement can be identified as a rare clinical feature of the MRS disease, which is confirmed difficult to be managed.

## Introduction

Melkersson-Rosenthal syndrome (MRS) is a rare oro-facial granulomatosis, which is clinically characterized by recurrent oro-facial swelling, relapsing facial palsy and a fissured tongue. Melkersson-Rosenthal Syndrome was first described in 1928 as peripheral facial paralysis and swelling. In 1931, Rosenthal identified the third sign which is the fissured tongue [1,2]. Herein, we report a case of a 29-year-old woman with an unusual presentation of MRS that had been misdiagnosed for three years.

## Patient and observation

**Patient information:** a 29-year-old married woman was referred to the department of internal medicine with complaints of recurrent swelling of the lips and gingival enlargement. The patient received nifedipine as an antihypertensive therapy after being diagnosed with hypertension.

**Clinical findings:** three years before her admission to our department, she had experienced an acute peripheral facial nerve palsy quickly treated with corticostoids for over two weeks. After receiving corticostoids, she improved and the symptom had disappeared. Two years later, she presented the second episode of facial paralysis. She had been treated by corticosteroids, injection of botulic toxin and facial workout but she kept permanent sequelae of facial paralysis.

**Figure 1:**
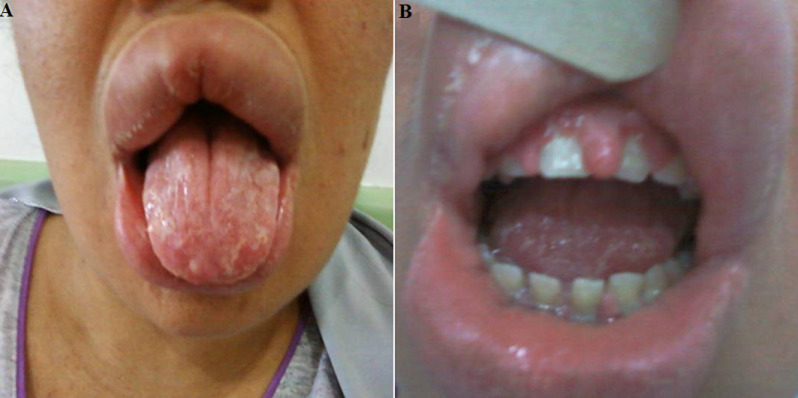
A) swelling of the lips with geographic tongue; B) gingival hyperplasia

**Timeline of current episode:** the last episode was when she was pregnant. She had experienced a recurrent swelling at her lips. On her clinical examination, she had a right facial palsy, a diffuse swelling mainly in the upper lip, deep fissures grooves on the dorsal surface of the tongue (Figure 1A) and finally a gingival hyperplasia (Figure 1B). The rest of the physical exam was unremarkable.

**Figure 2:**
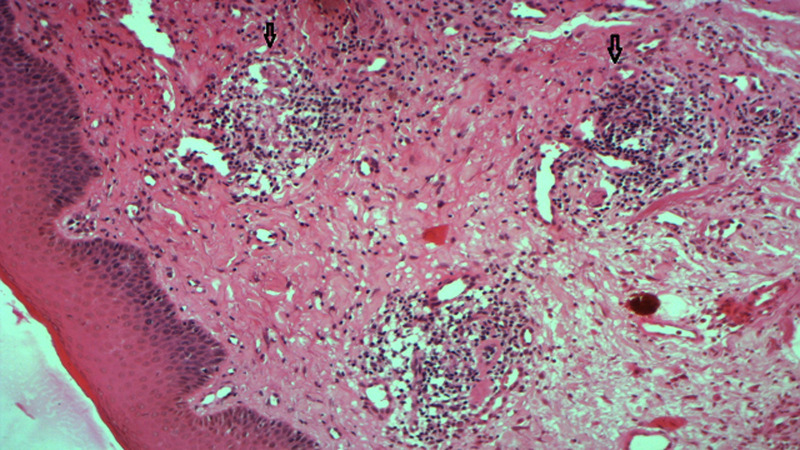
the biopsy of the swollen lip showing granulomatous cheilitis characterized by perivascular lymphocytic infiltration with granuloma formation (haematoxylin-eosin, original magnification x 100; granuloma near dilated vessel

**Diagnostic assessment:** no significant laboratory findings were present: the liver enzymes and the renal function were within the normal levels. The calcium and phosphate levels were respectively 2.46 mmol/l and 1mmol/l and there was no hypercalciuria. The complement fractions were normal and the antinuclear antibodies were negative. Histopathological examination of a sampling removed from the upper lip showed focal perivascular infiltration composed of lymphoplasmocytes cells, dermal edema and granuloma formation near dilated vessels (Figure 2). Accordingly, the diagnosis of sarcoidosis was first evoked, but there was diffusional interstitial lung disease or mediastinal lymph node enlargement on chest radiography. In addition, the histopathological examination of salivary glands indicated that there is no granulomas and the angiotensin-converting enzyme level was normal.

**Diagnosis:** the diagnosis of MRS was based on both the clinical presentation of the classical triad and the compatible.

**Therapeutic interventions:** the patient had been treated with high dose of prednisone (1 mg/Kg/day) during four weeks followed by a slow tapering. As for the gingival enlargement, it was removed with scalpel gingivectomy. The histopathological examination of the gingival sampling confirmed the granulomatosis infiltration.

**Figure 3:**
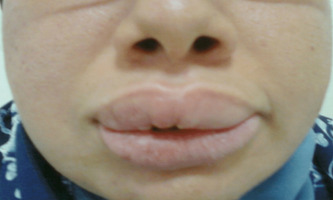
after treatment, residual swelling of the lips

**Follow-up and outcome of interventions:** two weeks following discharge, the patient had residual facial edema and facial palsy with the persistence of the fissured tongue (Figure 3). Immunosuppressant therapy was planned in case of further relapses.

**Patient perspective:** I have an expectation of being cured with the prescribed treatment. Therefore, that to continue the treatment and the regular follow-up.

**Informed consent:** the patient gave informed consent.

## Discussion

Melkersson-Rosenthal syndrome is a rare disease characterized by three cardinal symptoms which are orofacial swelling, facial paralysis and the fissured tongue [3]. The classical triad of MRS is observed only in 8% to 18% of the patients aged between 25 and 40 years [4,5]. The most common clinical feature of MRS is the orofacial edema. Often misdiagnosed as angioedema, the orofacial edema is localized mainly at the upper lip [6]. Facial paralysis is seen in 50% to 60% of patients with MRS [6]. As in our case, it has been observed that facial palsy may manifest on the patient many years before being diagnosed with MRS. The least common clinical feature of MRS is the fissured tongue which is seen in 30-35 % of the cases [6].

The clinical examination of our patient shows another presenting symptom of the disease which is the gingival enlargement. To the best of our knowledge, 13 cases out of a series of 30 were reported to have granulomatous gingival enlargement. Therefore, we may conclude that the granulomatous gingival manifestation of MRS can be considered as an exceptional feature of the disease. Six patients of the latter series underwent a gingival biopsy. The result of the biopsies analysis showed the characteristic microscopic lesions of MRS in five of them whereas the remaining biopsy showed heavy plasma cell infiltration in the connective tissue [7]. In 1989, Knychalska-Karwan reported another case of Hyperplasia of gingivae in the course of incomplete form of Melkersson-Rosenthal syndrome [8].

Remaining a disease of unknown etiology, the diagnosis and management of the MRS is still difficult. According to previous researches, genetic factors may be involved in the formation of the disease. Moreover, allergy and viral infections have been reported to be causative factors as well [3]. It should be pointed out that MRS may be present either by its own or in association with other chronic diseases such as sarcoidosis and Crohn disease [6]. Histological examination has been suggested to be necessary in case of uncompleted presentation as it helps determining the characteristic aspect of the disease, which is the non-caseous perivascular granulomas [6].

Given its unknown etiology, there have been no effective medical treatments for MRS up to the present time. However, authors recommend as treatments systemic steroid at the first line therapy with short courses or intra-lesional steroid administration to avoid side effects of this drug [3]. The combination of dapsone with local steroid therapy was reported to be safe and effective therapy [9]. Other treatment choices have been tried as well for their anti-inflammatory or immunomodulatory effects such as topical tacrolimus, oral thalidomide, dapsone, and doxycycline. Nevertheless, these treatments have not been found to be always sufficient [3]. In case of resistance, Stein *et al*. recommended anti-tumor necrosis factor, an agent which was proved to be successful in treating a patient with non-caseating granulomatous cheilitis, neurological impairment and elevated immunoglobulin. This patient improved with adalimumab therapy after the failure of steroids, azathioprine, and methotrexate [10]. Antibiotics with their anti-inflammatory function like minocycline, were also used in cases of granulomatous cheilitis [6,11]. The cheiloplasty reduction is possible when the lesions are fixed and not evolving [6]. Facial nerve decompression may become necessary for patients with recurrent facial paralysis. The management of gingival hyperplasia is difficult as well, and gingivectomy was recommended in this situation. Nonetheless, recurrences and exacerbations of the gingival swellings are still possible [7].

## Conclusion

Melkersson-Rosenthal syndrome is a rare multidisciplinary entity of unknown pathogenesis. One of the most significant findings to emerge from our case report is that gingival enlargement should be considered as a rare clinical feature of the MRS disease. To date, there is no known effective treatment. Corticosteroid remains the first line therapy to relieve orofacial edema. Surgical treatment choice is recommended in case of gingival hyperplasia but relapses are still possible.
